# *IGFBP3* As a Potential Biomarker and Therapeutic Target in Hepatocellular Carcinoma: A Multi‐Cohort Analysis

**DOI:** 10.1002/jcla.70265

**Published:** 2026-07-06

**Authors:** Yin Tao, Yunji Xu, Xupeng Chen, Yali Zhou, Jingli Fu

**Affiliations:** ^1^ Department of General Surgery Zhuzhou Central Hospital Zhuzhou Hunan China; ^2^ Department of General Surgery The Second Affiliated Hospital, Hengyang Medical College, University of South China Hengyang Hunan China; ^3^ Department of Clinical Laboratory Zhuzhou Central Hospital Zhuzhou Hunan China; ^4^ Department of Neonatology Zhuzhou Central Hospital Zhuzhou Hunan China; ^5^ Department of Infectious Diseases Zhuzhou Central Hospital Zhuzhou Hunan China

**Keywords:** biomarker, hepatocellular carcinoma, *IGFBP3*, immune infiltration, prognosis

## Abstract

**Objective:**

This study aimed to investigate the expression patterns and prognostic significance of insulin‐like growth factor‐binding protein 3 (*IGFBP3*) in hepatocellular carcinoma (HCC) and other cancer types.

**Methods:**

*IGFBP3* expression was analyzed using The Cancer Genome Atlas (TCGA)‐LIHC, International Cancer Genome Consortium (ICGC‐LIRI‐JP), Gene Expression Omnibus (GEO; GSE102079 and GSE112790), and Genotype‐Tissue Expression (GTEx) databases. Experimental validation included immunohistochemistry (IHC) and quantitative real‐time PCR (qRT‐PCR) on 10 paired HCC tissues. Survival was assessed via Kaplan–Meier analysis in TCGA, ICGC, and 47‐patient cohorts stratified by *IGFBP3* immunoreactive score (IRS). Univariate and multivariate Cox regression identified prognostic factors. Immune infiltration correlations were evaluated using ESTIMATE and TIMER algorithms, with pan‐cancer analysis conducted via TCGA database.

**Results:**

*IGFBP3* was significantly downregulated in HCC across all cohorts and validated in our cohort (*p* < 0.01). Elevated intratumoral *IGFBP3* correlated with advanced tumor stage, high grade, and elevated AFP. High *IGFBP3* predicted poorer overall survival in TCGA (HR = 1.666, *p* = 0.004) and ICGC (HR = 4.631, *p* = 0.0009), and was associated with shorter survival in our cohort (*p* = 0.0002). Multivariate analysis confirmed *IGFBP3* as an independent prognostic factor (HR = 2.95, *p* = 0.014). IGFBP3 expression positively correlated with immunosuppressive cell infiltration (M2 macrophages, regulatory T cells) and enriched pathways including TGF‐β and JAK–STAT signaling. Pan‐cancer analysis revealed cancer‐specific expression patterns, with HCC showing both overall downregulation and high‐risk prognostic value in high‐expression tumors.

**Conclusion:**

*IGFBP3* was low expression in HCC, yet high expression indicates poor prognosis. This suggests that *IGFBP3* is a potential novel prognostic biomarker and therapeutic target in HCC.

## Graphical abstract

This study demonstrates that insulin‐like growth factor‐binding protein 3 (IGFBP3) exhibits a dual role in hepatocellular carcinoma (HCC), being significantly downregulated in tumor tissues compared to normal liver, yet its high expression within tumors is strongly associated with advanced disease and poor prognosis. Multi‐cohort analysis and experimental validation confirm that elevated intratumoral IGFBP3 correlates with aggressive clinicopathological features, immunosuppressive microenvironment infiltration, and serves as an independent prognostic biomarker. These findings position IGFBP3 as a context‐dependent biomarker and a potential therapeutic target in advanced HCC.

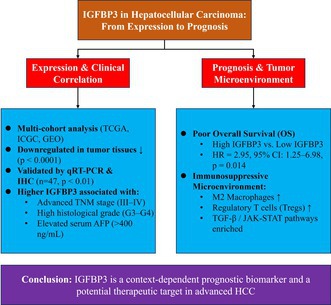

## 1 Introduction

Hepatocellular carcinoma (HCC) is a significant global health concern owing to its aggressive nature and poor prognosis [1]. It is the fourth leading cause of cancer‐related mortality worldwide, with approximately 905,700 new cases and 830,200 deaths reported annually [2]. Key risk factors for HCC include chronic HBV/HCV infection, metabolic disorders (e.g., NAFLD and diabetes), excessive alcohol consumption, and exposure to environmental toxins [3, 4, 5]. The incidence of HCC varies significantly between regions [6]. Its aggressive biology, coupled with frequent late‐stage diagnosis, culminates in a dismal prognosis [7, 8], with the 5 year survival rate for advanced disease remaining below 20% [2, 9]. Despite the advent of systemic therapies, including tyrosine kinase inhibitors and immune checkpoint inhibitors, which have modestly improved median survival, long‐term efficacy is severely hampered by almost universal tumor recurrence and the development of drug resistance [10, 11]. This stark clinical reality underscores the urgent need to decipher the molecular drivers of HCC progression and therapeutic failure.

Tumor heterogeneity and a complex network of dysregulated signaling pathways underpin the therapeutic recalcitrance of HCC [12, 13, 14, 15]. Among these, the insulin‐like growth factor (IGF) axis is critically implicated in promoting hepatocarcinogenesis, driving proliferation, metastasis, and conferring resistance to therapy [16, 17]. However, direct targeting of core components like IGF‐1R has yielded limited clinical success, highlighting the complexity of this pathway and the necessity to explore alternative nodal points for intervention [17].

Insulin‐like growth factor‐binding protein 3 (*IGFBP3*), a key modulator of the IGF axis, has emerged as a protein of significant interest due to its context‐dependent duality in cancer biology [18]. It can function as a classic tumor suppressor by sequestering IGF ligands and inhibiting canonical Wnt/β‐catenin signaling [18, 19]. Conversely, IGF‐independent, pro‐oncogenic roles of *IGFBP3*, potentially through interaction with other cell surface receptors, have also been documented, suggesting a complex role in tumor‐stroma interactions and disease progression [20, 21]. Despite its biological plausibility, the clinical and prognostic significance of *IGFBP3* expression in HCC remains ambiguous and insufficiently characterized. Previous studies have reported conflicting associations with patient outcomes, and a comprehensive analysis integrating multi‐omics data with validation in well‐defined clinical cohorts is lacking [21, 22]. This ambiguity significantly impedes the evaluation of *IGFBP3* as a reliable biomarker or a viable therapeutic target.

This study aims to comprehensively elucidate the clinical and prognostic significance of *IGFBP3* in HCC by integrating bioinformatic analyses with experimental validation. First, we analyzed *IGFBP3* expression, clinical correlations, and prognostic significance using the TCGA‐LIHC, ICGC‐LIRI‐JP, and GEO (GSE102079 and GSE112790) datasets. Subsequently, to validate these findings in a clinical context. Furthermore, we explored potential mechanisms by investigating the correlation between IGFBP3 expression and immune cell infiltration as well as enriched pathways. Finally, we performed a pan‐cancer analysis to examine the expression and prognostic significance of *IGFBP3* across various tumor types.

## 2 Materials and Methods

### 2.1 Data Collection

**FIGURE 1:**
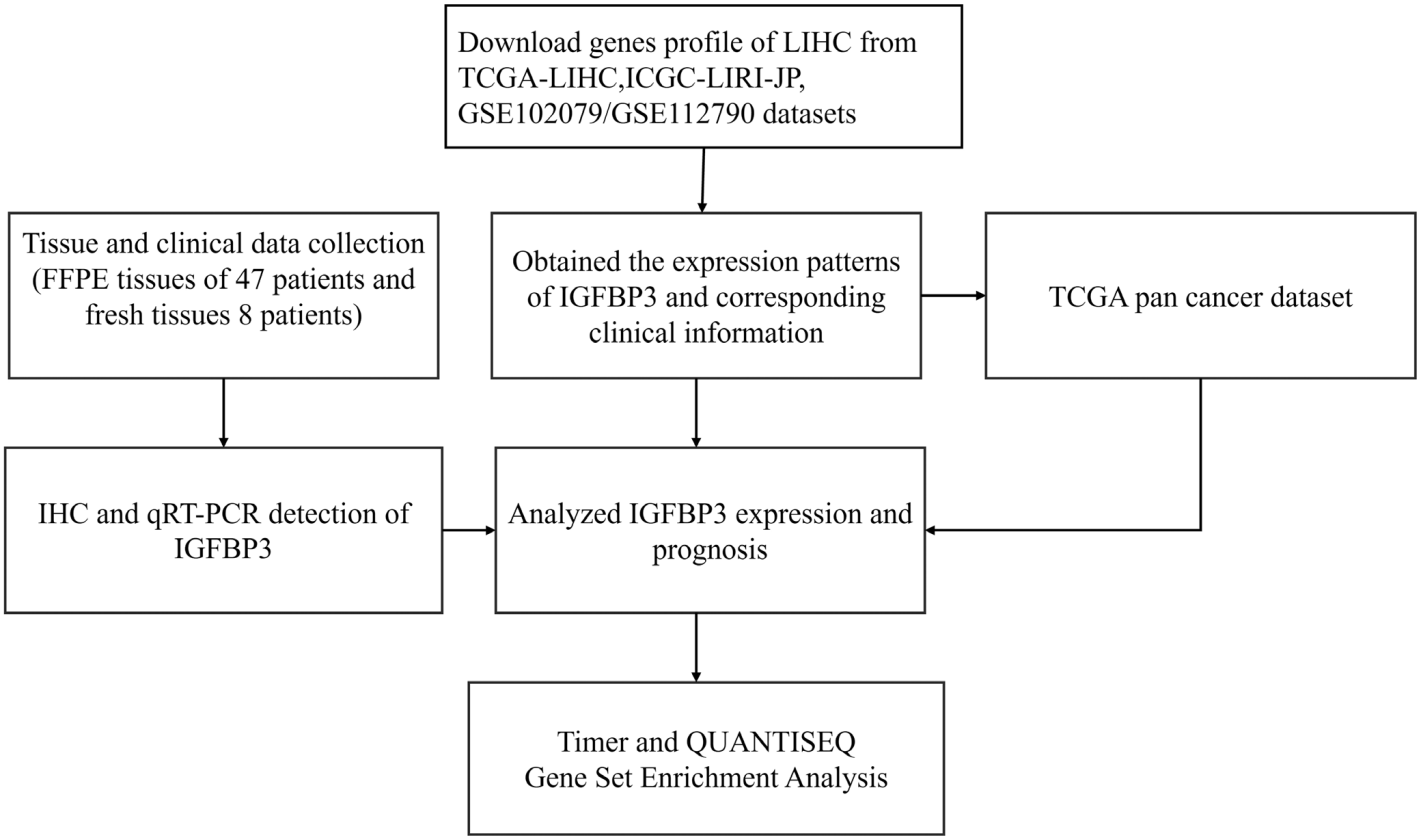
Schematic representation of the study workflow.

IGFBP3 transcriptome data and clinical information for HCC were retrieved from four independent cohorts: (1) The Cancer Genome Atlas (TCGA‐LIHC; https://portal.gdc.cancer.gov), (2) International Cancer Genome Consortium‐LIRI‐JP (ICGC‐LIRI‐JP; https://dcc.icgc.org), and (3) Gene Expression Omnibus (GEO, https://www.ncbi.nlm.nih.gov/geo/) datasets (GSE102079 and GSE112790). Additionally, pan‐cancer *IGFBP3* expression profiles (36 cancer types) and overall survival (OS) data were extracted from TCGA (https://www.cancer.gov/ccg/research/genome‐sequencing/tcga). The workflow of this study is illustrated in Figure 1. Additionally, pan‐cancer *IGFBP3* expression profiles (36 cancer types) and overall survival data were extracted from TCGA for a descriptive overview.

### 2.2 Analysis of IGFBP3 Expression Patterns and Survival Parameters

RNA‐seq raw count data and clinical annotations for HCC were downloaded from TCGA, ICGC, and GEO platforms using TCGAbiolinks (v3.14.1) and GEOquery (v2.66.0). Samples with missing *IGFBP3* expression values or incomplete survival records (OS < 30 day) were excluded. *IGFBP3* expression levels were normalized using transcripts per million (TPM) and stratified into high and low groups using the median as a cutoff value. Prognostic significance was assessed using Kaplan–Meier survival analysis with log‐rank tests in TCGA and ICGC cohorts.

### 2.3 Immune Cell Infiltration Analysis

#### 2.3.1 Timer

The abundance of immune cell subsets in TCGA‐LIHC tumor samples was quantified using TIMER 2.0, a web platform (http://timer.cistrome.org/) [23]. Bulk RNA‐seq data (FPKM normalized and log2‐transformed) from TCGA‐LIHC were analyzed using TIMER's constrained least‐squares regression model to deconvolute six immune cell types: B cells, CD4+ T cells, CD8+ T cells, neutrophils, macrophages, and dendritic cells. Tumor purity was automatically adjusted using TIMER's pre‐computed models to minimize confounding effects. The correlation between *IGFBP3* expression (log2‐transformed TPM values) and immune cell infiltration levels was evaluated using Spearman's rank correlation, with significance set at *p* < 0.05. All immune cell infiltration analyses are based on computational deconvolution of bulk RNA‐seq data, which provides estimates of relative immune cell abundance but cannot definitively distinguish closely related subsets (e.g., M1 vs. M2 macrophages) or establish causal relationships. Tumor purity was automatically adjusted by TIMER using pre‐computed models to minimize confounding effects. Results from these analyses should be interpreted as correlative evidence generating hypotheses for future experimental validation.

#### 2.3.2 QUANTISEQ

The quanTIseq computational framework [24] was used to estimate the absolute immune cell densities (cells/mm^2^) in TCGA‐LIHC samples. Raw RNA‐seq reads (FASTQ files) were aligned to the GRCh38 reference genome using STAR v2.7.10a, and gene‐level counts were generated using featureCounts v2.0.3. Expression matrices were normalized using the trimmed mean of M‐values (TMM) method to correct for compositional biases. The quanTIseq deconvolution model, which uses linear least‐squares regression with immune cell‐specific signature matrices (including M1/M2 macrophages and regulatory T cells), was executed using quanTIseq R package. To validate the immune cell estimates, IHC results from matched LIHC tissue sections were compared with computational predictions.

### 2.4 Tissue Collection

Two independent patient cohorts from Zhuzhou Central Hospital were included. A primary cohort comprised 47 patients with HCC (treated between January 2020 and December 2022), from whom formalin‐fixed, paraffin‐embedded (FFPE) tumor and adjacent non‐tumor tissues were retrieved for immunohistochemistry. Additionally, a separate cohort of 10 treatment‐naïve patients (recruited between August and October 2025) was enrolled. Paired fresh‐frozen HCC and adjacent tissues (≥ 2 cm from the tumor margin) were collected from these patients for molecular analysis, including quantitative real‐time PCR (qRT‐PCR) and immunohistochemistry (IHC). All samples were pathologically confirmed. The study was approved by the hospital's Institutional Review Board (KY2025110‐01), and written informed consent was obtained from all participants in accordance with the Declaration of Helsinki.

Clinical data were collected from electronic medical records, including age, gender, serum alpha‐fetoprotein (AFP) level, tumor size, TNM stage (AJCC 8th edition), histological grade, vascular invasion, and overall survival (OS). OS was defined as the time from surgery to death from any cause or the last follow‐up.

### 2.5 qRT‐PCR and IHC

qRT‐PCR: Total RNA was extracted using TRIzol (Invitrogen, #15596026) and reverse‐transcribed into cDNA using the PrimeScript RT Master Mix (Takara, #RR036A). Amplification was conducted on a QuantStudio 5 Real‐Time PCR System (Thermo Fisher). The following primer sequences were used: for the internal control GAPDH, forward 5‐GGAAGCTTGTCATCAATGGAAATC‐3 and reverse 5‐TGATGACCCTTTTGGCTCCC‐3; for the target gene *IGFBP3*, forward 5‐AGACACACTGAATCACCTGAAGT‐3 and reverse 5‐AGGGCGACACTGCTTTTTCTT‐3. The relative expression of *IGFBP3* was calculated using the 2 − ΔΔCT method.

IHC: Formalin‐fixed paraffin‐embedded sections were subjected to antigen retrieval (citrate buffer, pH 6.0) and blocked with 10% goat serum. The slides were incubated overnight with anti‐*IGFBP3* antibody (Abcam, #ab313875; 1:200), followed by incubation with horseradish peroxidase (HRP)‐conjugated secondary antibody and DAB visualization. ImageJ (v1.53) was used to calculate the integrated optical density (IOD) normalized to the tissue area.

### 2.6 Immunohistochemistry (IHC) and Evaluation

IHC staining was evaluated independently by two experienced pathologists who were blinded to the clinical data. The staining intensity was scored as follows: 0 (negative), 1 (weak), 2 (moderate), and 3 (strong). The percentage of positive tumor cells was scored as follows: 0 (< 5%), 1 (5%–25%), 2 (26%–50%), 3 (51%–75%), and 4 (> 75%). The final immunoreactivity score (IRS) was calculated by multiplying the intensity score by the percentage score, resulting in a range from 0 to 12. The median IRS value was used as the cut‐off to classify patients into low (IRS ≤ 4) and high (IRS > 4) IGFBP3 expression groups.

### 2.7 Statistical Analysis

Statistical analyses were performed using R software (version 4.3.1) and SPSS Statistics (version 26.0). Continuous variables were compared using the Student's *t*‐test for normally distributed data or the Mann–Whitney U test for non‐normally distributed data. Survival differences were analyzed using the log‐rank test with Kaplan–Meier curves. Independent prognostic factors were identified through univariate and multivariate Cox proportional hazards regression, with results reported as hazard ratios (HR) and 95% confidence intervals (CI). A two‐tailed *p* < 0.05 was considered significant, with false discovery rate (FDR) correction applied for multiple comparisons. The proportional hazards assumption for Cox regression models was assessed using the Schoenfeld residual test. In addition to the median‐based dichotomization of *IGFBP3* expression for Kaplan–Meier visualization, we also evaluated its prognostic significance as a continuous variable using Cox regression to avoid potential bias from arbitrary cutoff selection.

## 3 Results

### 3.1 Downregulation of *IGFBP3* in HCC

**FIGURE 2:**
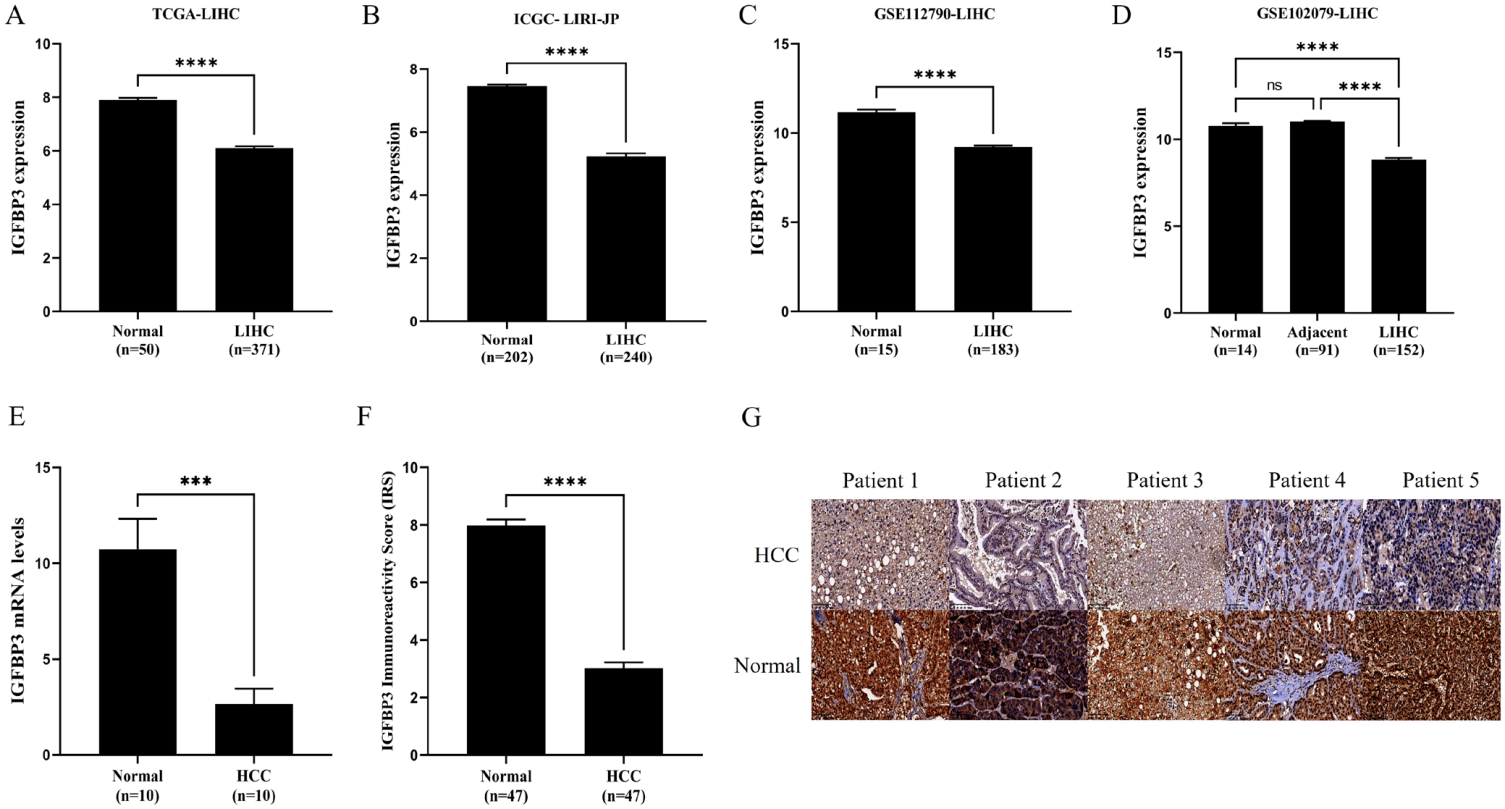
Downregulation of IGFBP3 in HCC across multiple cohorts. (A‐D) *IGFBP3* transcript levels are significantly lower in HCC than in normal liver across four independent datasets (TCGA, ICGC, GSE102079, GSE112790). (E) Validation of *IGFBP3* mRNA downregulation by qRT‐PCR in eight paired fresh‐frozen samples. (F‐G) IGFBP3 protein expression is markedly reduced in HCC tissues, as shown by representative IHC staining and quantification of immunoreactivity scores in 47 paired samples. (***p < 0.001; ****p < 0.0001).

The expression of *IGFBP3* was significantly reduced in HCC compared to that in normal liver tissue, as evidenced by multiple datasets: TCGA‐LIHC, ICGC‐LIRI‐JP, GSE102079, and GSE112790 (all *p* < 0.0001, Figure 2A–D). This downregulation was further validated at the protein level in our independent clinical cohort of HCC. qRT‐PCR analysis of eight paired fresh‐frozen samples corroborated this finding, showing significant downregulation of IGFBP3 mRNA in HCC (*p* < 0.01, Figure 2E). Immunohistochemistry (IHC) revealed that IGFBP3 protein, primarily localized in the cytoplasm, was markedly reduced in tumor tissues (median Immunoreactivity Score [IRS] = 3) versus adjacent non‐tumor tissues (median IRS = 7; *p* < 0.0001, Figure 2F,G).

### 3.2 Correlation of *IGFBP3* Expression With Clinical Classification of HCC

**FIGURE 3:**
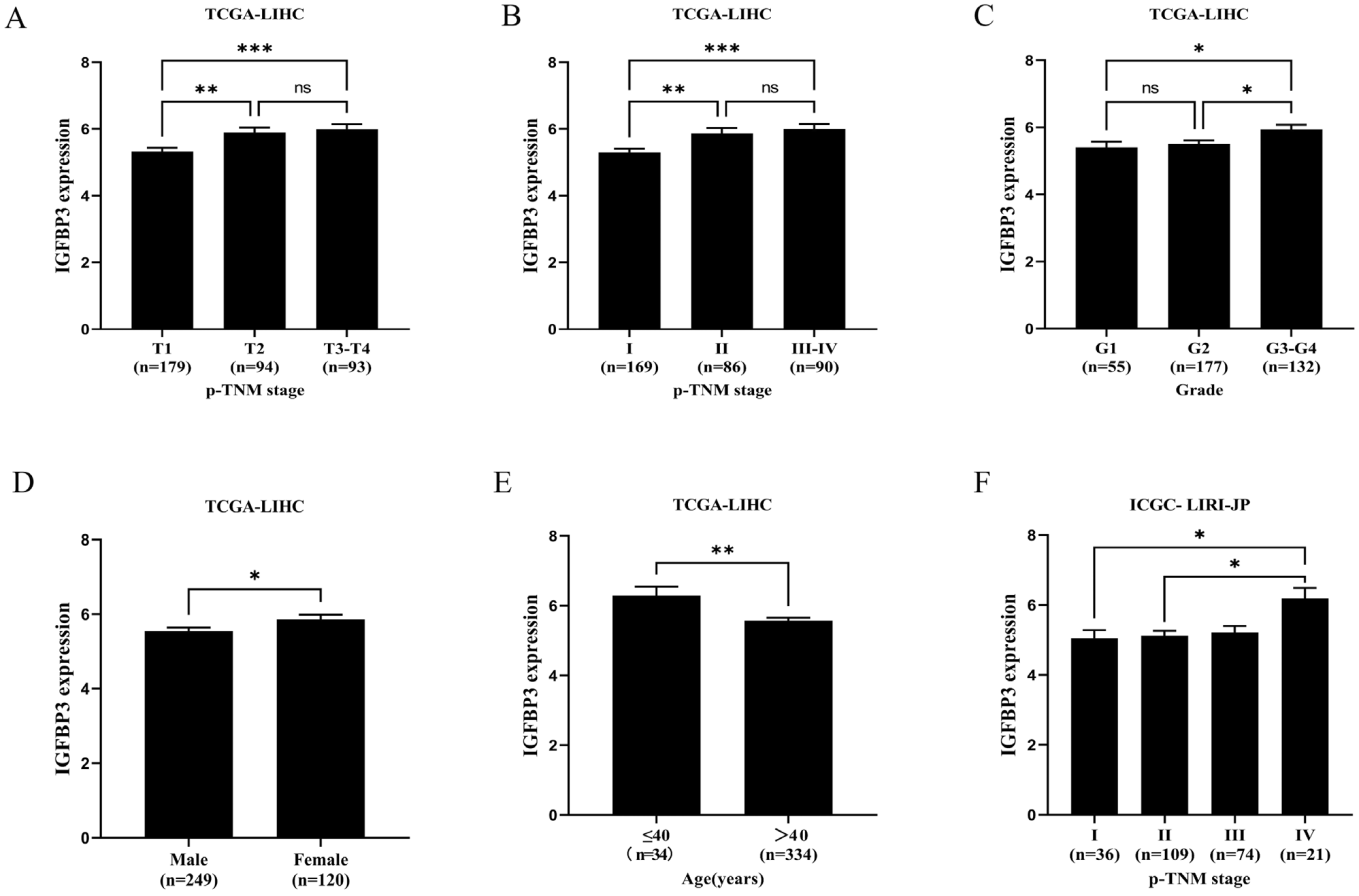
Association of IGFBP3 expression with clinical classification in HCC. (A‐E): IGFBP3 expression shows significant associations with age, gender, histological grade, and tumor stage in the TCGA‐LIHC cohort, respectively. (F): In the ICGC‐LIRI‐JP cohort, IGFBP3 expression significantly correlates with tumor stage.

The correlation between *IGFBP3* expression and various clinical parameters were by analyzed. In the TCGA‐LIHC dataset, *IGFBP3* expression was significantly elevated in advanced tumor stages (III‐IV) compared to earlier stages (Stage I vs. II: *p* < 0.01; Stage I vs. III‐IV: *p* < 0.001, Figure 3A) and in higher T stages (T1 vs. T2: *p* < 0.01; T1 vs. T3‐T4: *p* < 0.001, Figure 3B). Elevated *IGFBP3* expression was also observed in high‐grade tumors (G3‐G4) versus lower grades (G1 vs. G3‐G4: *p* < 0.05; G2 vs. G3‐G4: *p* < 0.05, Figure 3C). Furthermore, *IGFBP3* expression was significantly lower in male patients and in patients aged > 40 years (*p* < 0.05 and *p* < 0.01, respectively. Figure 3D,E). These findings were partially validated in the ICGC‐LIRI‐JP cohort, where elevated *IGFBP3* expression was confirmed in stage IV HCC relative to stage I and stage II tumors (Stage I vs. IV: *p* < 0.05; Stage II vs. IV: *p* < 0.05; Figure 3F). This association was confirmed in our cohort of 47 patients stratified by the median IRS (cut‐off = 4), where high *IGFBP3* expression was significantly associated with elevated serum AFP (> 400 ng/mL; *p* = 0.035), advanced TNM stage (III–IV; *p* = 0.008), and higher tumor grade (G3–G4; *p* = 0.022). No significant associations were found with age, gender, tumor size, or vascular invasion (Table 1).

**TABLE 1:** Association between IGFBP3 expression and clinicopathological characteristics in 47 HCC patients.

Characteristic	Category	Total (*n* = 47)	IGFBP3 low (*n* = 24)	IGFBP3 high (*n* = 23)	*p*
Age (years)	≤ 60/> 60	30/17	17/7	13/10	0.401
Gender	Male/Female	32/15	18/6	14/9	0.371
AFP (ng/mL)	≤ 400/> 400	25/22	16/8	9/14	0.035
Tumor size (cm)	≤ 5/> 5	29/18	16/8	13/10	0.573
TNM stage	I–II/III–IV	28/19	18/6	10/13	0.008
Histological grade	I–II/III–IV	30/17	19/5	11/12	0.022
Vascular invasion	No/Yes	31/16	18/6	13/10	0.237

### 3.3 Prognostic Significance of *IGFBP3* Expression in HCC

**FIGURE 4:**
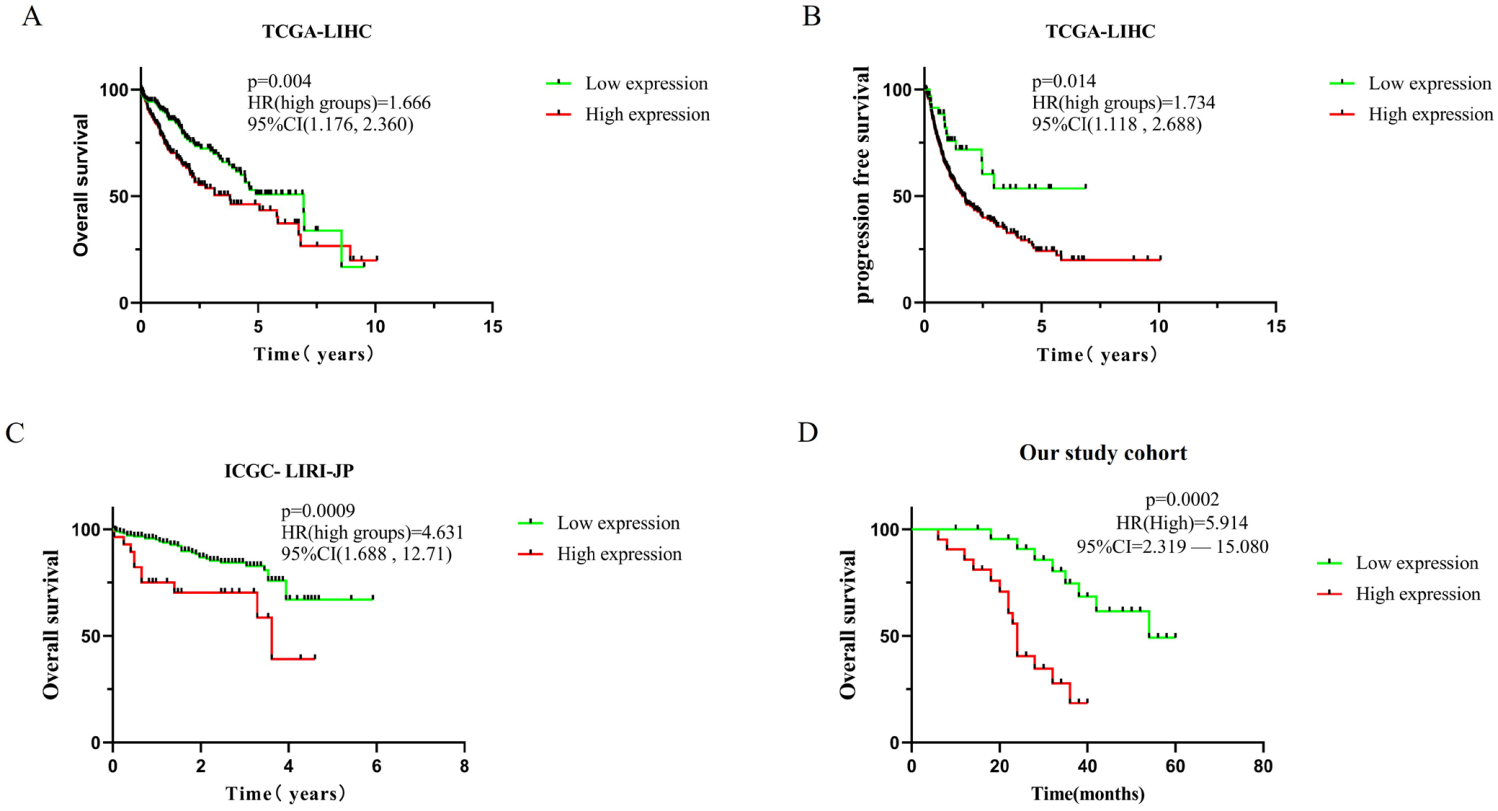
Prognostic value of IGFBP3 expression in HCC. (A, B): Kaplan–Meier curves comparing overall survival (OS) and progression‐free survival (PFS) between HCC patients with high vs. low IGFBP3 expression in the TCGA‐LIHC cohort. (C): Kaplan–Meier curve comparing overall survival (OS) between HCC patients with high vs. low IGFBP3 expression in the ICGC‐LIRI‐JP cohort. (D): Kaplan‐Meier curve comparing overall survival (OS) between HCC patients with high vs. low IGFBP3 expression in our independent clinical cohort of 47 patients (log‐rank *p* = 0.0002).

The adverse prognostic impact of high *IGFBP3* expression was consistently observed across multiple cohorts. In public datasets, patients with high *IGFBP3* expression had significantly worse survival outcomes. Specifically, in the TCGA‐LIHC cohort, high expression predicted poorer overall survival (OS: HR = 1.666, *p* = 0.004, Figure 4A) and progression‐free survival (PFS: HR = 1.734, *p* = 0.014, Figure 4B). Similarly, in the ICGC‐LIRI‐JP cohort, high *IGFBP3* was associated with markedly reduced OS (HR = 4.631, *p* = 0.0009, Figure 4C).

Critically, this prognostic association was robustly validated in our independent clinical cohort of 47 patients. Kaplan–Meier analysis confirmed that patients with high *IGFBP3* expression had a significantly shorter median overall survival (24 months) compared to those with low expression (38 months; log‐rank *p* = 0.0002, Figure 4D). Univariate Cox regression identified high *IGFBP3* expression (HR = 3.10, *p* = 0.008, Table 2), advanced TNM stage, and high histological grade as significant predictors of poor OS. Most importantly, multivariate Cox analysis demonstrated that high *IGFBP3* expression remained an independent prognostic factor for worse OS after adjusting for TNM stage and histological grade (HR = 2.95, 95% CI: 1.25–6.98, *p* = 0.014, Table 2).

**TABLE 2:** Univariate and multivariate Cox regression analyses of overall survival in 47 HCC patients.

Variable	Univariate analysis HR (95% CI)	*p*	Multivariate analysis HR (95% CI)	*p*
Age (> 60 vs. ≤ 60)	1.25 (0.51–3.05)	0.628		
Gender (Male vs. Female)	1.18 (0.46–3.04)	0.732		
AFP (> 400 vs. ≤ 400 ng/mL)	1.89 (0.80–4.45)	0.145		
Tumor size (> 5 vs. ≤ 5 cm)	1.65 (0.69–3.94)	0.257		
TNM stage (III–IV vs. I–II)	3.12 (1.28–7.58)	0.012	2.61 (1.05–6.50)	0.039
Histological grade (G3–G4 vs. G1–G2)	2.78 (1.15–6.73)	0.023	2.25 (0.91–5.55)	0.079
Vascular invasion (Yes vs. No)	1.72 (0.70–4.21)	0.237		
IGFBP3 expression (High vs. Low)	3.10 (1.35–7.12)	0.008	2.95 (1.25–6.98)	0.014

To avoid potential bias introduced by dichotomization, we further analyzed *IGFBP3* as a continuous variable using univariate Cox regression in the TCGA‐LIHC cohort. Continuous *IGFBP3* expression remained significantly associated with worse overall survival (HR = 1.09, 95% CI: 1.04–1.15, *p* = 0.002). The proportional hazards assumption was tested using the Schoenfeld residual test and was satisfied (global *p* > 0.05).

### 3.4 Correlation of *IGFBP3* Expression With Immune Cell Infiltration and Enriched Pathways in HCC

**FIGURE 5:**
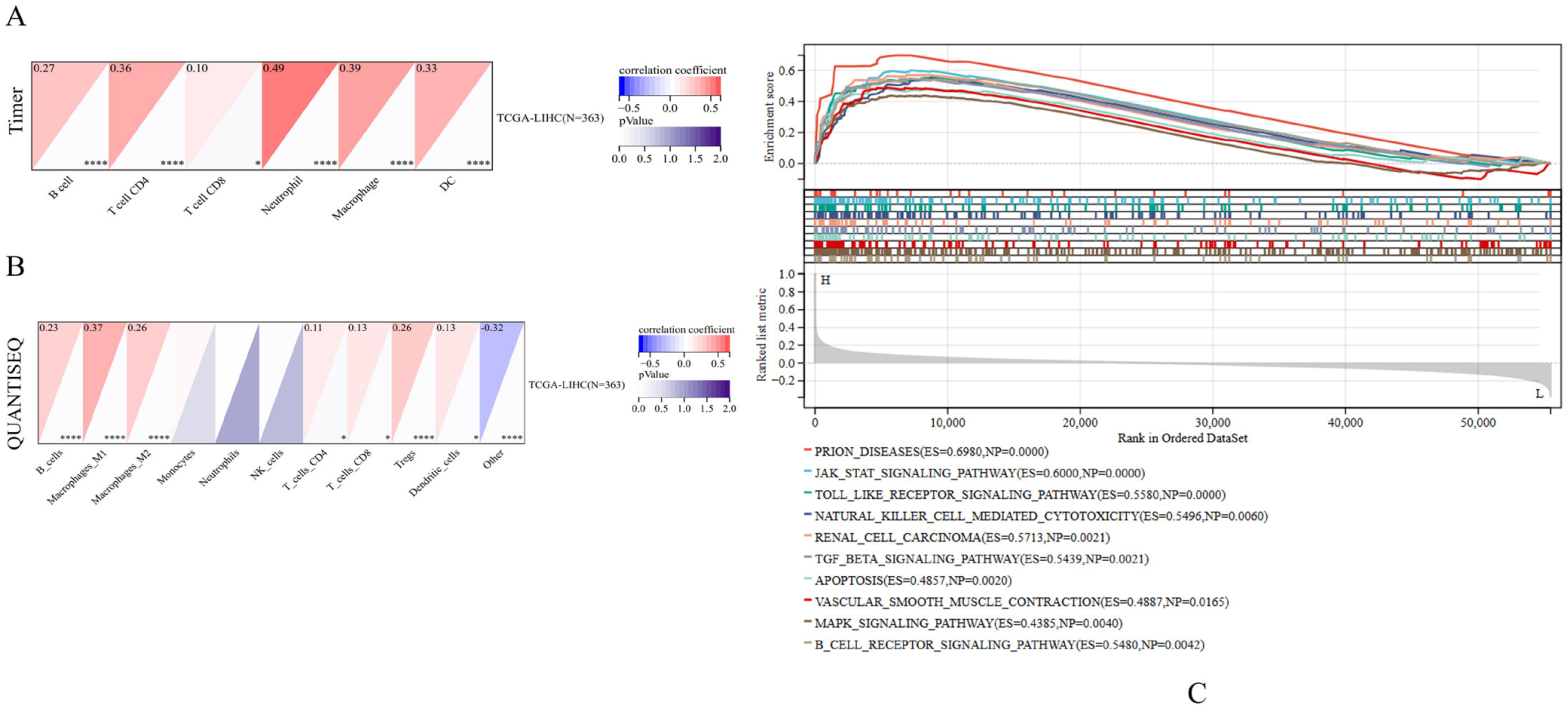
Association of IGFBP3 expression with immune infiltration and pathway enrichment in HCC. (A, B): Correlation between IGFBP3 expression and immune cell infiltration levels in the TCGA‐LIHC cohort analyzed using TIMER and QUANTISEQ algorithms. (C): Gene Set Enrichment Analysis (GSEA) of hallmark pathways associated with IGFBP3 expression in TCGA‐LIHC.

Analysis of the TCGA‐LIHC dataset revealed significant correlations between IGFBP3 expression and immune infiltration. The TIMER algorithm showed strong positive correlations (*p* < 0.0001) with neutrophils (*r* = 0.49), macrophages (*r* = 0.39), CD4+ T cells (*r* = 0.36), dendritic cells (*r* = 0.33), and B cells (*r* = 0.27), and a moderate correlation with CD8+ T cells (*r* = 0.10, *p* < 0.05) (Figure 5A). Further subset analysis via QUANTISEQ confirmed positive correlations with M1 (*r* = 0.37) and M2 macrophages (*r* = 0.26), B cells (r = 0.23), and regulatory T cells (*r* = 0.26) (all *p* < 0.0001), and moderate positive correlations with CD4+ T cells, CD8+ T cells, and dendritic cells (*r* = 0.11–0.13, *p* < 0.05). A negative correlation was observed with “Other cells” (r = −0.32, *p* < 0.0001) (Figure 5B). These correlative findings suggest a potential association between IGFBP3 expression and an immunosuppressive microenvironment, but causal inference requires experimental validation.

**FIGURE 6:**
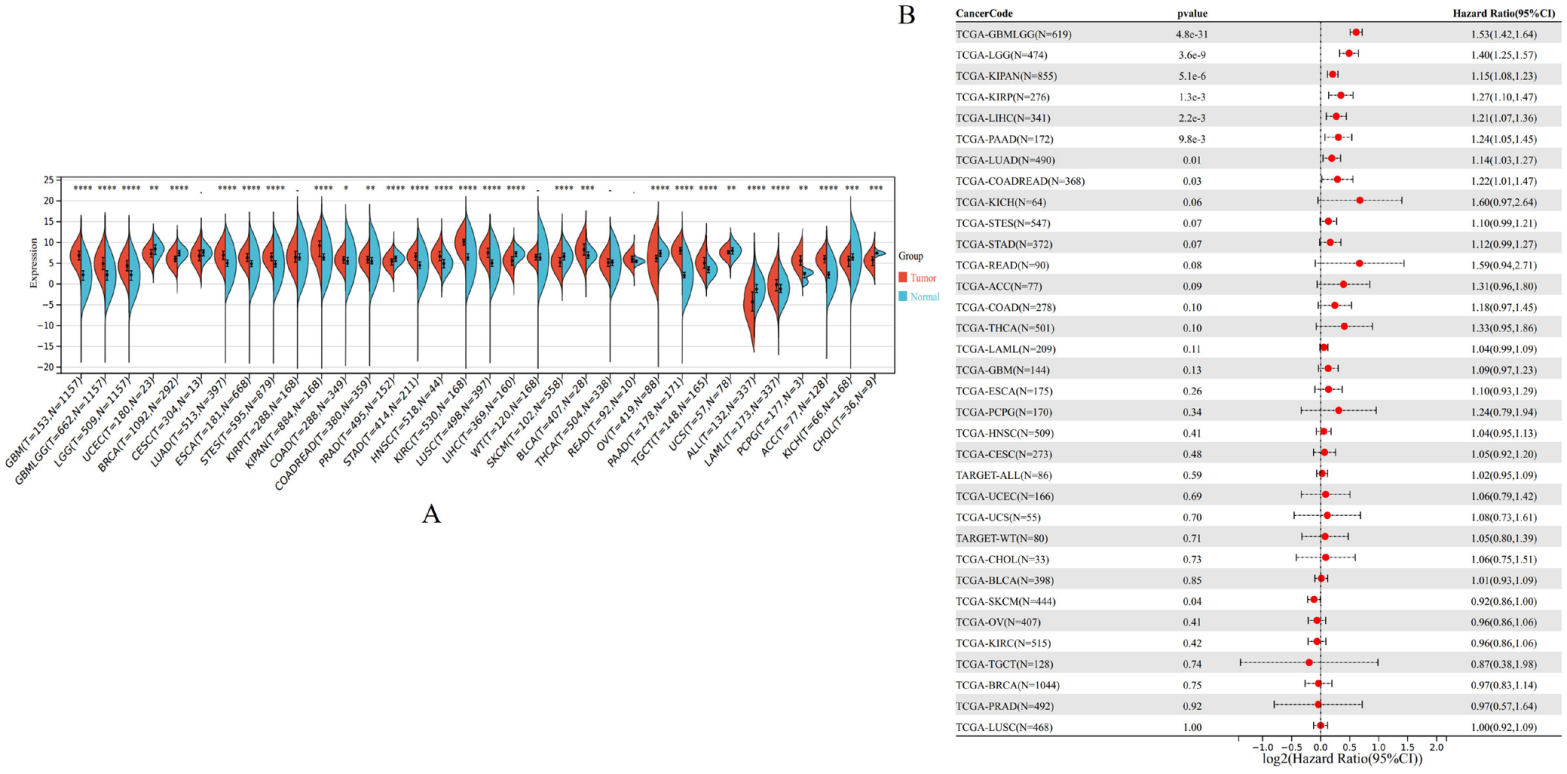
Pan‐cancer analysis of IGFBP3 expression and its prognostic significance (A, B): Evaluation of IGFBP3 expression levels and their association with prognosis across the 36 cancer types listed in the GTEx and TCGA databases.

Gene Set Enrichment Analysis (GSEA) identified multiple pathways significantly enriched in association with *IGFBP3* expression (all *p* < 0.05), including JAK–STAT signaling, Toll‐like receptor signaling, natural killer cell mediated cytotoxicity, TGF‐beta signaling, apoptosis, MAPK signaling, and B cell receptor signaling pathway (Enrichment Scores: 0.44–0.70) (Figures 5C and 6).

### 3.5 Pan‐Cancer Analysis of *IGFBP3* Expression and Prognostic Significance

To provide a broad context for our findings in HCC, we performed a pan‐cancer analysis of *IGFBP3* expression and its prognostic significance across 36 cancer types using TCGA data (complete results are presented in Figure S1A and Table S1). Expression patterns varied considerably across cancer types. Notably, HCC exhibited a distinct pattern: *IGFBP3* expression was significantly downregulated compared to normal liver tissue, yet higher expression within HCC tumors was associated with poor prognosis (HR = 1.21, 95% CI: 1.07–1.36, *p* = 0.0022; Figure S1B and Table S2). This unique coexistence of overall downregulation and high‐risk prognostic value underscores the context‐dependent role of *IGFBP3* in HCC.

## 4 Discussion

Hepatocellular carcinoma (HCC) is characterized by profound molecular heterogeneity and limited response to current therapies, underscoring the need for biomarkers that reflect its complex biology [12, 13, 14, 15]. In this study, we identified *IGFBP3* as a molecule exhibiting a stage‐dependent dual role in HCC pathogenesis and prognosis. Our study demonstrates that although *IGFBP3* expression is systematically downregulated in HCC tissues compared to normal liver tissues [25] consistent with a potential tumor‐suppressive role—its elevated expression within the tumor microenvironment, seen in a subset of HCC patients, is paradoxically associated with advanced disease and serves as a strong independent predictor of poor prognosis [26]. Thus, the comparison between “normal vs. tumor” addresses early transformation events, while the comparison between “IGFBP3‐low vs. IGFBP3‐high within tumors” identifies a more aggressive, immune‐suppressive subgroup. This apparent contradiction suggests that *IGFBP3* function is context‐dependent, shifting from a potential tumor‐suppressive role in early hepatocarcinogenesis to a tumor‐promoting role in established, aggressive HCC [21, 22].

The tumor‐suppressive role of IGFBP3, primarily through sequestering IGF ligands and inhibiting the PI3K/AKT and MAPK pathways, is well‐documented in several cancers [18, 27, 28, 29]. Our observation of its significant downregulation in HCC aligns with this paradigm and suggests that *IGFBP3* loss may be a permissive event in early liver tumorigenesis. However, our clinical correlation analyses reveal a critical shift: in established HCC, higher intratumoral *IGFBP3* expression is linked to aggressive features, including advanced TNM stage, high histological grade, and elevated serum AFP. This pattern was consistently observed across the TCGA, ICGC, and our independent cohort. Importantly, multivariate analysis confirmed high *IGFBP3* expression as an independent risk factor for shorter overall survival.

Similar dual functions have been reported in other malignancies. For instance, in prostate [30, 31] and breast cancers [32, 33], *IGFBP3* can exhibit tumor‐suppressive effects in early stages but is often associated with treatment resistance and poor prognosis in advanced disease, potentially through mechanisms involving interaction with different signaling pathways or components of the tumor microenvironment. This conserved pattern across cancer types suggests that *IGFBP3* may be a more universal molecular switch, whose functional outcome is critically determined by the contextual cues of the evolving tumor ecosystem. Our findings in HCC thus contribute to and reinforce this emerging paradigm, highlighting *IGFBP3* as a compelling target for context‐aware therapeutic strategies [20, 22].

Our integrated bioinformatics and histopathological analyses provide mechanistic clues for the adverse prognostic role of high *IGFBP3*. GSEA revealed that IGFBP3‐high tumors are enriched in pathways driving metastasis and immunosuppression, notably TGF‐β signaling and JAK–STAT signaling. This suggests that in advanced HCC, *IGFBP3* may facilitate epithelial‐mesenchymal transition and stromal activation, thereby promoting tumor progression [21].

Most notably, our immune deconvolution analysis uncovered a significant positive correlation between IGFBP3 expression and the infiltration of multiple immune cell types, particularly immunosuppressive subsets such as M2 macrophages and regulatory T cells (Tregs), alongside a broad spectrum of other cells including neutrophils and dendritic cells. This association between IGFBP3 and an immunosuppressive microenvironment aligns with findings in other solid tumors. In colorectal and gastric cancers, IGFBP3 has been implicated in promoting the polarization of macrophages toward an M2 phenotype and recruiting Tregs, thereby facilitating tumor progression and immune evasion [33, 34, 35, 36]. While our data indicate IGFBP3's role in immune cell recruitment, the specific enrichment of these immunosuppressive subsets (M2, Tregs) likely fosters a microenvironment that effectively dampens anti‐tumor immunity, despite the co‐presence of some effector cells like M1 macrophages [37]. We therefore propose that in the context of advanced HCC, elevated IGFBP3 does not simply reflect a loss of its growth‐suppressive function; rather, it may actively contribute to an immunosuppressive niche—characterized by M2‐polarized macrophages and Treg recruitment—that dampens anti‐tumor immunity and facilitates immune evasion. This immunomodulatory axis provides a data‐supported, though still correlative, explanation for the poor survival outcomes in patients with high IGFBP3‐expressing HCC.

Our pan‐cancer analysis further contextualizes the complexity of IGFBP3, confirming its expression and prognostic impact are highly cancer‐type specific. Strikingly, HCC emerges as a distinct model where significant downregulation and high‐risk prognostic value coexist. This duality underscores that IGFBP3's function is not intrinsically oncogenic or suppressive but is dictated by the tissue and tumor microenvironmental context [21, 38]. Advanced liver organoid models may help dissect this context‐dependent duality in future studies [39].

### 4.1 Limitations and Future Directions

This study has limitations. First, the sample size of our institutional cohort (*n* = 47) is relatively modest, which may limit statistical power and precision. Therefore, this cohort should be viewed as complementary protein‐level validation of the prognostic association, rather than the primary evidence. The primary prognostic conclusion is derived from the large TCGA‐LIHC (*n* = 365) and ICGC‐LIRI‐JP (*n* = 231) cohorts, where the sample sizes are adequate and the results are highly consistent. Sensitivity analyses including a parsimonious multivariate model and calculation of the events‐pervariable ratio (6.7) suggested that the multivariate result in our cohort was not driven by overfitting. Nevertheless, larger independent cohorts are needed to confirm the optimal cutoff‐ and generalizability of *IGFBP3* as a prognostic biomarker. Second, the functional validation of the proposed mechanisms, particularly the causal role of IGFBP3 in shaping the immunosuppressive niche, requires further investigation using in vitro and in vivo models, such as IGFBP3‐knockdown or overexpression in HCC cell lines and mouse models. Additionally, the spatial relationship between IGFBP3‐expressing tumor cells and specific immune infiltrates needs clarification via multiplex immunohistochemistry or spatial transcriptomics. Third, our immune infiltration analyses rely on computational deconvolution of bulk RNA‐seq data (TIMER and quanTIseq), which have inherent limitations. These tools estimate relative immune cell abundance but cannot definitively distinguish closely related subsets (e.g., M1 vs. M2 polarization states) and are influenced by tumor purity and transcriptional covariates. Most importantly, the observed correlations between IGFBP3 expression and immune cell subsets do not imply causation. Therefore, our findings should be interpreted as hypothesis‐generating. Future studies are required to establish causal relationships, for example using in vitro co‐culture systems of HCC cells with immune cells following IGFBP3 manipulation, or spatial transcriptomics to resolve cell–cell interactions within the tumor microenvironment.

Our multi‐cohort integrative study reveals IGFBP3 as a dynamic, stage‐dependent regulator in HCC. It transitions from a potential guardian lost in early transformation to a biomarker of aggressive, immune‐suppressive advanced disease. Unraveling the molecular switch that reprograms IGFBP3 function during HCC progression will be crucial for developing stage‐specific diagnostic and therapeutic strategies.

## 5 Conclusion

This study reveals a stage‐dependent duality of IGFBP3 in hepatocellular carcinoma (HCC). While globally downregulated in tumor versus normal tissue, elevated intratumoral IGFBP3 expression is significantly associated with advanced disease (stage III–IV, grade G3–G4) and serves as an independent prognostic marker of poor survival. Mechanistically, high IGFBP3 correlates with an immunosuppressive tumor microenvironment, characterized by enriched M2 macrophage and regulatory T‐cell infiltration. These findings position IGFBP3 as a context‐dependent biomarker and a potential therapeutic target in advanced HCC.

## Author Contributions

Yin Tao and Yunji Xu contributed equally to this work as co‐first authors. Yin Tao: Conceptualization, Methodology, Formal analysis, Investigation, Validation, Visualization, Writing Original Draft, Writing Review and Editing. Yunji Xu: Conceptualization, Methodology, Software, Data curation, Formal analysis, Investigation, Validation, Visualization, Writing Original Draft, Writing Review and Editing. Jingli Fu: Conceptualization, Funding acquisition, Resources, Supervision, Corresponding author. Xupeng Chen: Resources, Investigation, Validation. Yali Zhou: Resources, Investigation, Validation. All authors reviewed and approved the final version of the manuscript.

## Funding

This study was supported by the Health Research Project of Health Commission of Hunan Province (grant number: D202304017818, 20255423). Hunan Provincial Natural Science Foundation (No. 2025JJ81027).

## Ethics Statement

The study was approved by the hospital's Institutional Review Board (KY2025110‐01), and written informed consent was obtained from all participants in accordance with the Declaration of Helsinki.

## Conflicts of Interest

The authors declare no conflicts of interest.

## Supporting information


**Figure S1:** Pan‐cancer analysis of IGFBP3 expression and its prognostic significance (A‐B): Evaluation of IGFBP3 expression levels and their association with prognosis across the 36 cancer types listed in the GTEx and TCGA databases.
**Table S1:** The expression of IGFBP3 in Pan‐Cancer.
**Table S2:** The prognosis of IGFBP3 in Pan‐Cancer.

## Data Availability

All data used in this study can be obtained by contacting the corresponding author via e‐mail at 340468999@qq.com.
