# Supplementary material for: IGFBP3 As a Potential Biomarker and Therapeutic Target in Hepatocellular Carcinoma: A Multi‐Cohort Analysis
Source: J Clin Lab Anal. 2026 Jul 6;40(13):e70265. doi: 10.1002/jcla.70265 (PMC13371280; doi:10.1002/jcla.70265)
Supplement: Supplementary file 1 — Figure S1: Pan‐cancer analysis of IGFBP3 expression and its prognostic significance (A‐B): Evaluation of IGFBP3 expression levels and their association with prognosis across the 36 cancer types listed in the GTEx and TCGA databases. Table S1: The expression of IGFBP3 in Pan‐Cancer. Table S2: The prognosis of IGFBP3 in Pan‐Cancer. [file JCLA-40-e70265-s001.docx]

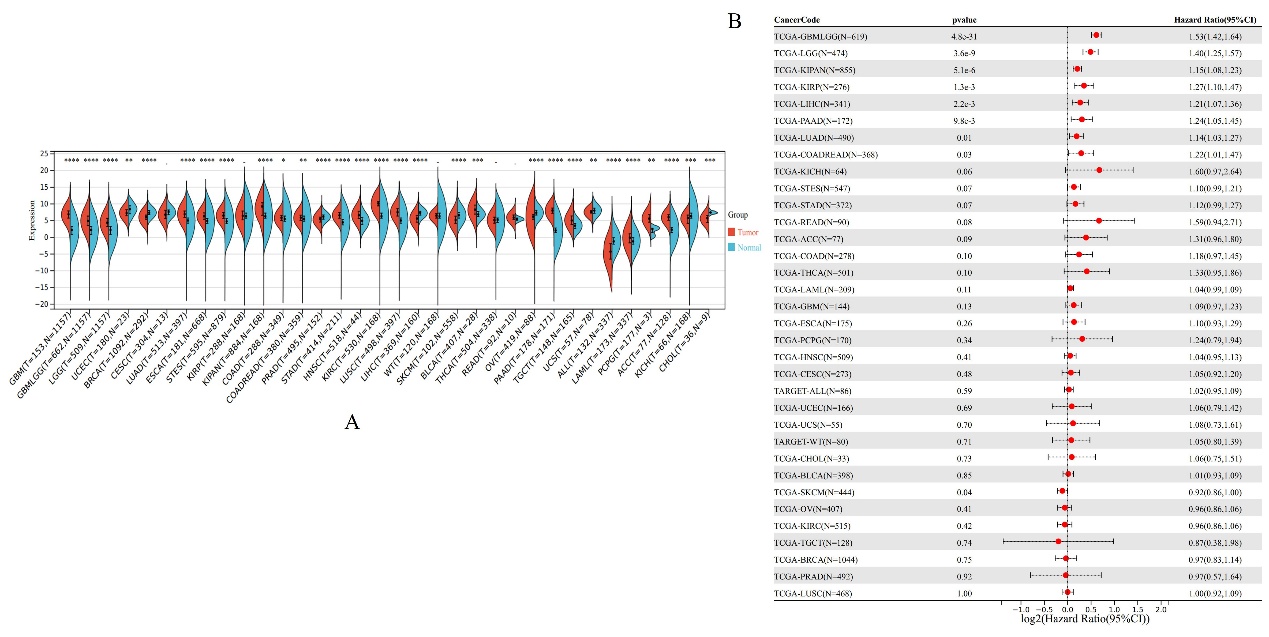


**Supplementary Figure 1: Pan-cancer analysis of IGFBP3 expression and its prognostic significance.** (A-B): Evaluation of IGFBP3 expression levels and their association with prognosis across the 36 cancer types listed in the GTEx and TCGA databases.

Supplementary Table 1, The expression of IGFBP3 in Pan-Cancer

| Cohort | Expression | | p |
| --- | --- | --- | --- |
|  | Tumor | Normal |  |
| TCGA-GBM | 6.78±1.71 | 2.05±1.84 | 2.0e-80 |
| TCGA-GBMLGG | 4.89±2.11 | 2.05±1.84 | 1.4e-137 |
| TCGA-LGG | 4.33±1.88 | 2.05±1.84 | 1.5e-93 |
| TCGA-LUAD | 6.93±1.43 | 5.03±1.32 | 1.3e-74 |
| TCGA-ESCA | 6.37±1.49 | 4.81±1.58 | 2.0e-32 |
| TCGA-STES | 6.50±1.32 | 4.72±1.64 | 7.7e-109 |
| TCGA-KIPAN | 8.48±2.42 | 6.27±1.78 | 1.3e-27 |
| TCGA-COAD | 5.80±1.21 | 5.46±2.03 | 0.01 |
| TCGA-COADREAD | 5.84±1.18 | 5.45±2.00 | 1.1e-3 |
| TCGA-STAD | 6.57±1.24 | 4.41±1.77 | 1.6e-57 |
| TCGA-HNSC | 6.70±1.53 | 5.00±1.61 | 5.3e-10 |
| TCGA-KIRC | 9.90±1.46 | 6.27±1.78 | 5.7e-70 |
| TCGA-LUSC | 7.53±1.62 | 5.03±1.32 | 8.0e-92 |
| TCGA-BLCA | 8.15±1.95 | 7.01±1.20 | 4.2e-4 |
| TCGA-PAAD | 7.83±1.43 | 2.15±1.86 | 8.9e-55 |
| TCGA-TGCT | 5.20±1.82 | 3.58±1.22 | 7.2e-16) |
| TCGA-LAML | -0.34±2.29 | -0.99±1.80 | 2.1e-6 |
| TCGA-PCPG | 5.64±1.71 | 2.01±1.33 | 5.2e-3 |
| TCGA-ACC | 5.86±1.35 | 2.18±1.60 | 1.6e-29 |
| TCGA-UCEC | 7.30±1.28 | 8.25±1.65 | 4.2e-3 |
| TCGA-BRCA | 6.04±1.00 | 7.43±0.94 | 3.0e-75 |
| TCGA-PRAD | 5.34±1.06 | 6.13±0.93 | 3.3e-15 |
| TCGA-LIHC | 5.65±1.47 | 7.23±0.94 | 2.1e-31 |
| TCGA-SKCM | 5.45±1.72 | 6.56±1.28 | 8.6e-13 |
| TCGA-OV | 6.15±1.42 | 7.36±1.35 | 5.6e-14 |
| TCGA-UCS | 7.51±0.97 | 8.05±1.15 | 9.5e-3 |
| TCGA-ALL | 4.68±3.64 | 0.99±1.80 | 1.2e-26 |
| TCGA-KICH | 5.42±1.71 | 6.27±1.78 | 3.3e-4 |
| TCGA-CHOL | 5.74±1.47 | 7.45±0.40 | 6.6e-4 |

**Supplementary Table 2, The prognosis of IGFBP3 in Pan-Cancer**

| Cohort | Hazard Ratio | p |
| --- | --- | --- |
| TCGA-GBMLGG | 1.53(1.42,1.64) | 4.8e-31 |
| TCGA-LGG | 1.40(1.25,1.57) | 3.6e-9 |
| TCGA-LUAD | 1.14(1.03,1.27) | 0.01 |
| TCGA-KIRP | 1.27(1.10,1.47) | 1.3e-3 |
| TCGA-KIPAN | 1.15(1.08,1.23) | 5.1e-6 |
| TCGA-COADREAD | 1.22(1.01,1.47) | 0.03 |
| TCGA-THYM | 2.36(1.24,4.49) | 5.9e-3 |
| TCGA-LIHC | 1.21(1.07,1.36) | 2.2e-3 |
| TCGA-MESO | 1.44(1.21,1.70) | 2.3e-5 |
| TCGA-PAAD | 1.24(1.05,1.45) | 9.8e-3 |
| TCGA-SKCM | 0.92(0.86,1.00) | 0.04 |
